# “What should be computed” for supporting post-pandemic recovery policymaking? A life-oriented perspective

**DOI:** 10.1007/s43762-021-00025-8

**Published:** 2021-11-19

**Authors:** Junyi Zhang, Tao Feng, Jing Kang, Shuangjin Li, Rui Liu, Shuang Ma, Baoxin Zhai, Runsen Zhang, Hongxiang Ding, Taoxing Zhu

**Affiliations:** 1 Mobilities and Urban Policy Lab, Graduate School of Advanced Science and Engineering, Hiroshima University, Higashihiroshima, Japan; 2 Department of the Built Environment, Eindhoven University of Technology, Eindhoven, The Netherlands; 3 College of Architecture and Urban Planning, Tongji University, Shanghai, China; 4 School of Economics and Management, Shijiazhuang Tiedao University, Shijiazhuang, China

**Keywords:** COVID-19 pandemic, Daily life, Life-oriented approach, Big data, Questionnaire data, Post-pandemic recovery, Policymaking

## Abstract

The COVID-19 pandemic has caused various impacts on people’s lives, while changes in people’s lives have shown mixed effects on mitigating the spread of the SARS-CoV-2 virus. Understanding how to capture such two-way interactions is crucial, not only to control the pandemic but also to support post-pandemic urban recovery policies. As suggested by the life-oriented approach, the above interactions exist with respect to a variety of life domains, which form a complex behavior system. Through a review of the literature, this paper first points out inconsistent evidence about behavioral factors affecting the spread of COVID-19, and then argues that existing studies on the impacts of COVID-19 on people’s lives have ignored behavioral co-changes in multiple life domains. Furthermore, selected uncertain trends of people’s lives for the post-pandemic recovery are described. Finally, this paper concludes with a summary about “what should be computed?” in *Computational Urban Science* with respect to how to catch up with delays in the SDGs caused by the COVID-19 pandemic, how to address digital divides and dilemmas of e-society, how to capture behavioral co-changes during the post-pandemic recovery process, and how to better manage post-pandemic recovery policymaking processes.

## Introduction

After the WHO declared the COVID-19 pandemic on March 11, 2020, the pandemic has caused more than 198,000,000 cumulative cases and more than 4,200,000 deaths across the whole world (Worldometers, 2021). Vaccination has been promoted globally at a slow pace, and considerable gaps exist across countries and continents. According to Our World in Data, by the end of July 2021, only 28% of the world population had received at least one dose of a COVID-19 vaccine; however, the share was only 1.1% in low-income countries (Our Word in Data, 2021). Major non-medical measures against the pandemic have relied heavily on partial or full lockdowns, restrictions on ‘non-essential’ activities, and working from home. Even with the above-mentioned efforts by all countries of the world, daily new cases by the end of July 2021 showed that a third global peak was emerging (Worldometers, 2021). It seems that restricting people’s daily activities have limited effects on preventing infections. Vaccinations have been observed to reduce deaths, but new infection cases still went up with the reopening of economic activities, even in those countries with a higher share of vaccinated people (e.g., the USA, the UK, Spain, France, and Israel). While the limited effects of the above measures may be related to the emergence of new variants, these observations suggest that *it is still unclear when the global society will return to a normal or a new normal state*.

More than seventeen agonizing months have already passed since the declaration of the COVID-19 pandemic. People’s lifestyles during the COVID-19 pandemic have been largely affected by government policy measures and individuals’ risk perceptions, which affected how people adjusted their daily life needs and subsequent activity-travel schedules and behaviors (Zhang, 2021a; Ding and Zhang, 2021). The current COVID-19 pandemic has become a ‘window of opportunity’ to promote more resilient and sustainable lifestyles, not only for general sustainable development but also in terms of preparing for future pandemics and other crises. During the first half of 2020, CO_2_ emissions were observed to decline significantly (Liu et al., 2020); however, the emissions had rebounded close to 2019 levels by the end of 2020 (Tollefson, 2021). *It still remains unclear whether or not changes in people’s lifestyles observed during the pandemic will be a long-term trend or as transient as “a fleeting cloud.”*

In addition to the above rebound effects of CO_2_ emissions, social exclusion issues should also be given attention. For example, online activities have helped a lot of people to keep safe from the infection on the one hand, while inaccessibility to online services has forced some groups of people, particularly low-income people, to live/work under a very risky environment, exacerbating existing social exclusion issues (more specifically, existing digital divide issues). Unemployment has been a serious global concern during the pandemic; however, policy makers in all countries have yet to find effective ways to lower the unemployment rate. The world’s largest 50 countries announced a total amount of USD14.6tn for pandemic-related spending in 2020, of which 13.0% was for long-term ‘recovery-type’ measures (UNEP, 2021). Within this 13.0%, only 18.0% was directed to green recovery initiatives. *It is crucial that governments build back better for a more inclusive and greener recovery; however, this involves various dilemmas.*

### Why ask the question “what should be computed?”

For the post-pandemic recovery, it is crucial to better manage people’s mobilities in terms of spatiotemporal trips and activities, changes in migration and residential locations, etc. In this regard, *Computable Urban Science* should play a key role in supporting the post-pandemic recovery policymaking. Accordingly, it is important to answer the question “what should be computed?” This study argues that it is important to transform people’s lives to promote the post-pandemic recovery and attempts to answer the above question based on a life-oriented approach (Zhang, 2017). The approach argues that people’s decisions in various life domains (e.g., migration, residence, daily activities and trips, time use and expenditure, social networking, health) are interdependent. Such interdependencies further suggest that analyses of a certain life domain should not be conducted without considering its connections with other life domains. The ignorance of such interdependencies may not only lead to biased understanding of a target life domain in theory; more importantly, it may further result in misleading policy decisions in practice.

### Chapter structure

**Fig. 1:**
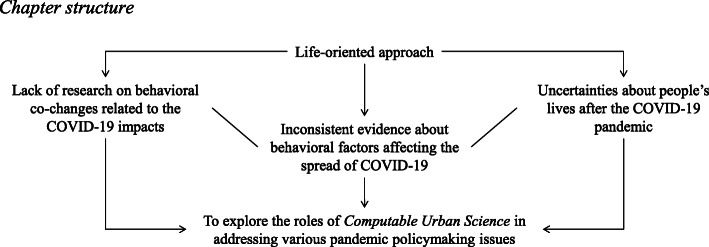
Research framework

The remaining part of this paper is organized based on the research framework shown in Fig. 1. First, inconsistent evidence about behavioral factors affecting the spread of COVID-19 is presented, aiming to reveal biased assessments on these factors, which have been major barriers to effective evidence-based COVID-19 policymaking. Second, impacts of COVID-19 on people’s lives are briefly reviewed, in order to figure out key behavioral issues leading to unsuccessful cross-sectoral collaboration. Third, uncertain trends of people’s lives for the post-pandemic recovery are discussed by showing results from a worldwide expert survey, illustrating key concerns about policymaking, and uncovering uncertain behavioral changes, where a new scientific view of planetary health is especially emphasized. Finally, by reflecting the findings from the above three contents, this paper concludes by summarizing “*what should be computed?*”

## Inconsistent evidence about behavioral factors affecting the spread of COVID-19

In the early stages of the pandemic, governments across the world developed various unprecedented policy interventions (e.g., travel restrictions, social distancing) to limit virus transmission and mitigate detrimental effects of the pandemic, which led to dramatic changes in people’s daily lives at a global scale. However, there are inconsisencies in the evidence on how the changed behaviour in human mobility and activities in open spaces helped to prevent infections, and how urban settings like population density and connectivity are associated with the spread of COVID-19.

### Mobility and travel behavior

Population movement and transportation infrastructure that increase inter- and intra-urban connectivity are considered to be key factors contributing to the spread of infectious diseases, as shown in reviews of existing literature about previous disease outbreaks (e.g., Ebola and SARS) (Connolly et al., 2020). This was also confirmed in the spread of COVID-19. Cartenì et al. (2020) found that the number of daily certified cases of COVID-19 infections is strongly linked to the trips made 21 days before the infection was confirmed. As such, restrictions on human movement can significantly limit the spread of the virus (Kraemer et al., 2020; Tian et al., 2020). More specifically, reductions in population flows (Fang et al., 2020), school closures (Litvinova et al., 2019), public transportation disruptions (Godzinski and Castillo, 2019), restriction of travelers from high-incidence locations (Milusheva, 2016), and working from home (Fadinger & Schymik, 2020) can significantly mitigate virus transmissions.

As shown in the literature, different transportation modes are linked with different resilience and transmission risks during the COVID-19 pandemic. One common indication is that non-motorized and individual transportation systems are more resilient to pandemics. For example, Zhang et al. (2020) found that the frequency of air flights and high-speed train (HST) services out of Wuhan not only increased the risk of infection of travelers, but also significantly increased the number of infected people in the destination cities. In turn, COVID-19 also produced some double-edged and structural effects on travel behavior and people’s mobility, through both forced and voluntary policies restricting movements. The positive outcome is that total travel declined, and people chose to bike and walk more. Teixeira and Lopes (2020) focused on the operation of subway and bike share systems (BSS) during the COVID-19 outbreak in New York and found that the city’s BBS saw a lower decrease than the subway system (71% vs. 90%), which indicated a modal shift from subway to bike-sharing. The same conclusion was confirmed in Budapest, where cycling and bike-sharing witnessed the lowest decline in demand (23% and 2%, respectively), while the highest decline was seen in public transport (80%) (Bucsky, 2020). They concluded, therefore, that the BBS had proven more resilient than the subway system during the COVID-19 pandemic. The negative side effect is that the COVID-19 experience may increase negative attitudes towards public transportation and increase preferences for individual travel modes (Aloi et al., 2020; De Haas et al., 2020). Some researchers suggested that investment in such non-motorized and individual transportation systems (e.g., BBS) not only contributes to containing the spread of the virus but can also increase service accessibility and help maintain social distance on overly crowded transportation systems during emergency situations (Biswas, 2020).

### Use of open/public spaces

During the outbreak of COVID-19, restrictions on the use of public spaces and physical distancing have been key policy measures to reduce virus transmission. Half of the world’s population was asked to stay at home or restrict movements in public (Sandford, 2020). These health intervention measures transformed individual activity behavior, and fundamentally changed our relationship with public spaces. While there is a lack of empirical evidence on the effects of the design of streets and open/public spaces on the dynamics of the COVID-19 spread and associated response measures, there are some who argue that in order to facilitate effective physical distancing in times of pandemics, cities need to give higher priority to active transportation modes and open/public spaces (Barbarossa, 2020). For example, as cities push for active transit in the post-COVID-19 city, streets may need to be redesigned to better accommodate the needs of pedestrians and cyclists and to provide ample green and open spaces which meet the outdoor exercise and recreation demands of citizens (Honey-Rosés et al., 2020). Such reconfigurations may also provide opportunities to further integrate urban greenery into cities, thereby achieving additional health and climate adaptation co-benefits. They may also contribute to resilience against other stressors and adverse events (Sharifi, 2019).

Physical distancing measures during the pandemic may encourage the redesign of various urban spaces and transport systems. More open space will be required within urban areas. The roles of parks and river waterfronts will become more and more important in people’s daily lives. This means existing urban layouts will be changed accordingly. Although large scale changes may not be achievable within a short period of time, especially given physical constraints like the lifespan of existing infrastructures and limited urban spaces, the functional transformation may still be highly beneficial to the people.

### Density and COVID-19 infection

High population density tends to increase the frequency of face-to-face interactions, which in turn are considered to be potential hotspots for droplet transmissions (Jahangiri et al., 2020). It was observed that COVID infection rates (per head of population) was highest in large urban centers globally, including New York, London, and Paris in the early stages of the pandemic. This might be explained by the fact that maintaining social distance is more challenging in high-density population areas. The association between density and COVID-19 infections have been extensively discussed and confirmed in existing studies. In China, the very high-risk zones of COVID-19 infection in Beijing and Guangzhou tended to occur in areas with higher population densities (Ren et al., 2020). Qiu et al. (2020) focus on transmission rates in early stages of the COVID-19 pandemic and found that population density had a significant positive effect on transmission rates in the first phase (from January 19 to February 1, 2020). Cartenì et al. (2020) conducted a similar study in different Italian regions and found higher transmission rates in regions with higher population densities. Accordingly, they suggested that in the high-density cities, the stricter implementation of public health measures (e.g., stringent quarantines, city lockdowns, and local public health measures) and the sharing of inter-city resources were two possible measures for reducing social interactions.

However, inconsistent evidence on the association between density and COVID-19 infections has also been presented in the literature. In 913 metropolitan counties of the USA, one study did not find a strong positive correlation between COVID-19 infections and mortality rates and activity density (Hamidi et al., 2020). They think the possible reason for the insignificant relationship may be that density may facilitate the implementation of social distancing orders, due to better home delivery services as well as a higher perceived susceptibility to the threat, leading to more precautionary behaviors. This interpretation is supported by Gallup polls, where residents of dense places were found to be more likely to practice basic social distancing than their counterparts in suburban and exurban areas (Saad, 2020). Surprisingly, compared with sprawling areas, Hamidi et al. (2020) observed slightly lower virus-related mortality rates in high-density locations. A similar study conducted in the Netherlands also failed to find a significant positive relationship between county density and infection rate (Boterman, 2020). In China, Lin et al. (2020) first found that both the percentage of the population arriving from Wuhan and the population density had a significant relationship with the spread rate of COVID-19; however, when controlling the former variable, they found that population density was not significant for virus spread rate, even in metropolitan regions with a high population density.

### Connectivity and city size

High connectivity means a more convenient environment for human mobility, which was also found to have a significant effect on the spread of COVID-19. Christidis and Christodoulou (2020) assessed the risk of spreading COVID-19 outside China and found that air travel has a decisive role in the spread of COVID-19 at the global level. Few countries maintained a high degree of connectivity, while containing virus transmission successfully (Sun et al., 2021). The high connectivity between cities in China, particularly with Wuhan, is the primary factor of the rapid spread of the virus without substantial public health interventions (Wu et al., 2020). Hamidi et al. (2020) also identified connectivity as a high risk factor for COVID-19 in the USA, and suggested more emphasis on connectivity rather than density when explaining the transmission dynamics of the virus. In terms of COVID-19 incidence, urban geometry (i.e. building geometry, road network and greenspace) was found to have played a more significant role than socio-demographic characteristics (Kwok et al., 2021). Regarding city size, Stier et al. (2020) estimated the growth rates of COVID-19 in US cities and found a power-law scaling relationship to city population size. They emphasize that policymakers need to implement more aggressive protection measures (e.g., distancing policies) in larger cities. According to these findings, planners are recommended to keep advocating for compact forms of urban development rather than sprawling ones; the desirability of compact urban development has been widely confirmed (Connolly et al., 2020; Hamidi et al., 2020).

### Summary

It is difficult to deny that the above-revealed various inconsistencies have worsened the trust of policymakers in scientific evidence, as often observed in key politicians’ public speeches, which unfortunately led to inconsistent COVID-19 policymaking across countries of the whole world. Even though better risk communications between scientists and politicians can enhance the trust of politicians in scientific evidence, in reality, the COVID-19 pandemic has been addressed “primarily in terms of political regulation and concerns and only marginally as a scientific matter” (Crabu et al., 2021). Inconsistent evidence about COVID-19 is not useful to encourage people’s voluntary behavioral changes for mitigating the impacts of COVID-19 and controlling the pandemic, either. Inconsistent evidence further brought about many unknowns to reveal the various future uncertainties. To avoid such inconvenience of ignoring science in practice and effectively involve various stakeholders for addressing future uncertainties, it is crucial to present more consistent evidence via interdisciplinary efforts.

## Impacts of COVID-19 on people’s lives: lack of research on behavioral co-changes

### Impacts on short-term decision making: daily life

People’s lifestyles during the COVID-19 pandemic have been largely affected by policy measures and individuals’ risk perceptions, due to adjustments in daily life needs and resulting activity-travel schedules and behaviors (Zhang, 2017; Ding and Zhang, 2021). Many studies have been conducted with respect to remote-work, online study, travel frequency, family life, time use, energy consumption, social networking, and physical activities (Parady et al., 2020; Shamshiripour et al., 2020; Guzman et al., 2021; Pérez-Rodrigo et al., 2021; Zhang, 2017; Zhang et al., 2021).

### E-activity

E-activity has become a major alternative in many people’s life activity choice sets and has brought about changes in urban mobility. To avoid being infected, the ways of making social contact at various places have had to be modified (Latsuzbaia et al., 2020). Policy measures also target different places to change individuals’ daily routines (Hotle et al., 2020; Abouk et al., 2021). Home-based activities increased sharply during the pandemic compared with the pre-pandemic period. Many people have shifted from traditional office-working to teleworking, and online shopping has witnessed its apogee (Shamshiripour et al., 2020). For example, Mouratidis and Papagiannakis (2021) found that in Greece,^1^ substantial increases were reported with respect to the frequencies of teleconferencing (57% increase), video calls with family or friends (55% increase), online learning (53% increase), and telework (49% increase). Such dramatic increases in e-activities have also been observed in many other countries. With millions of people now staying inside and working at home, COVID-19 has highlighted the huge impact that housing has on people’s lives and wellbeing.

Remarkable increases in online activities have largely reduced transport demand (e.g., Beck and Hensher, 2020; Borkowski et al., 2021; Google, 2021; Shakibaei et al., 2020). For example, many individuals have shifted their travel mode choices from public transport to private cars and to active transport (i.e., cycling and walking) when engaging in out-of-home activities (De Haas et al., 2020). Other research indicated that it might completely change the number and types of out-of-home activities people perform and how people reach these activities (De Vos, 2020). The movement patterns and destination choice behaviors of leisure activities also changed, as tourists began to focus on local areas and simplified travel routes (Jeon and Yang, 2021), and explored areas with natural environments and lower population densities, such as rural tourism destinations, where they could be more active (Zhu and Deng, 2020). Factors affecting these various behavior changes have been investigated, such as policy interventions, personal knowledge, and risk perceptions (Ding and Zhang, 2021; Hotle et al., 2020; Lu et al., 2021; Zhang, 2017).

However, not everybody can benefit equally from e-activities. It brings *threats* together with *opportunities* to occupations. On the one hand, not all jobs can be done at home. For example, in the USA (Dingel and Neiman, 2020), shares of jobs that can be done at home vary between 4% and 83%, where the industries with a share larger than 50% include educational services (83%), professional, scientific and technical services (80%), management of companies and enterprises (79%), finance and insurance (76%), information (72%), and wholesale trade (52%). The corresponding shares in essential services are very low, such as health care and social assistance (25%), retail trade (14%), accommodation and food services (4%). On the other hand, digital divides are a great concern. For example, while 70% of EU citizens use the Internet at least once a week, about 20% of EU citizens have never used the Internet (European Commission, 2021). In Japan, more than 90% of people aged 13–69 can access the Internet; however, about 30% of people aged 70–79 and about 40% of people aged 80 and above cannot use the Internet.^2^ Despite the existence of these digital divides, emerging on-demand information platforms are offering more and more new job opportunities. For example, since April 2020, Amazon.com has hired more than 175,000 people (Amazon, 2020), and Uber Eats’ delivery revenue in 2020 doubled that in 2019, reflecting the remarkable growth of the delivery labor market (Sumagaysay, 2020). The share of e-commerce and the “delivery” economy increased two to five times faster in 2020 than before the epidemic.

### Household energy consumption

According to the IEA, the drop in energy demand (including transport energy demand) during the COVID-19 pandemic may result in a record annual decline in carbon emissions of almost 8%.^3^ On the other hand, a third of the global population had to stay at home because of lockdowns.^4^ According to a review about changes in electricity demand caused by lockdowns and stay at home orders (Krarti and Aldubyan, 2021), the UK observed a 15% reduction in all sectors but a 17% increase in the residential sector, while Argentina showed a 20% reduction in all sectors, a 32.4% reduction in the industrial sector and a 18.2% reduction in the commercial sector. In Australia, a 14% increase was found in the residential sector, but only a 1% increase for all sectors. In Texas, electricity demand increased by 32%. In Canada, the COVID-19 effects on residential energy consumption were only observed when lockdown measures were stricter. A continuous observation by the Renewable Energy World with respect to 113 homes (79 with solar and 34 without solar; 50 having Level 2 EV (electric vehicle) chargers) at Austin, Texas from 2017 found a maximal 20% increase of residential energy consumption in March 2020, in comparison with the same month in previous years. On the other hand, EV charging dropped dramatically (Hinson, 2020).

Before the pandemic, the impact of teleworking or telecommuting and e-shopping on energy consumption has been examind for several decades (Henry et al., 2020). Initially, the effects were limited in the transport sector because the physical commuting trips were replaced partially or completely. Later, discussions were extended to include office buildings and residential buildings. In general, a relatively larger body of research supports the view that telecommuting only marginally contributes to energy consumption reduction, while possibly increasing overall energy demand, especially when taking into account the telecommuting consequences of residential movements, increase in energy consumption in houses, and the lack of flexibility of office buildings in reducing energy supply according to occupancy. However, some studies do find a positive contribution of telecommuting to energy reduction. In addition, e-shopping is generally considered to increase energy consumption because of the transport of deliveries.

Because of the tiredness of adapting to the pandemic situation, people’s adapted lifestyles may bounce back to pre-pandemic ones. It is crucial to continuously monitor changes in household energy consumption.

### Unhealthy behaviors of healthy people

Repeated lockdowns, restrictions on daily travel and activities, and social distancing measures have forced many people to adapt their daily lives to the pandemic situation. It seems that such adaptations have various health impacts on people who are unrelated to being infected.

During the pandemic, about 40% of adults in the U.S. reported symptoms of anxiety or depressive disorder, 36% reported difficulty sleeping, 32% stated eating problems, 12% showed an increase in alcohol consumption or substance use, and 12% described worsening chronic conditions (Panchal, 2021). According to Simone et al. (2021), unhealthy eating behaviors reported included mindless eating and snacking, eating more, decreased appetite or food intake, and an increase in eating disorder symptoms. Such behaviors have led to extreme unhealthy weight control behaviors in about 8% of respondents, less extreme unhealthy weight control behaviors in more than 50% of respondents, and binge eating in 14% of respondents. Poor stress management, greater depressive symptoms and moderate or extreme financial difficulties are found to be influential in these unhealthy weight control behaviors.

Children have also suffered from the pandemic. A Canadian study (Moore et al., 2020) found that children and youth performed less physical exercise, spent less time on outdoor activities, sat longer and slept more during the pandemic. As a result, 95.2% of children (97.2% girls, 93.5% boys) and 99.4% of youth (99.2% girls, 99.5% boys) could not meet healthy movement behavior guidelines. According to a report by UNICEF (2020a), 27% of the adolescents and young people in Latin America and the Caribbean felt anxious, 15% were depressed, 46% were less motivated to participate in activities they usually enjoyed, 36% were less motivated to do regular housework, and 43% of the women felt pessimistic about the future in comparison to 31% of the men.

### Impacts on long-term decision making: migration, employment and education

The above short-term changes may have long-lasting effects on urban development, particularly when employment and housing associated with online activities and the use of buildings and spaces (both physical and virtual) are involved. Concerning long-term changes in people’s lifestyles, a worldwide expert survey revealed a variety of changes, including in activity participation from physical spaces to virtual spaces (e.g., online working, online shopping, online education), job choices in association with teleworking availability, residential relocation, migration to/from populated cities, and car dependence associated with less confidence in using public transport, and so on (Zhang et al., 2021).

*Migration*: Migration behavior is a key factor in determining the population of a city. In Tokyo, Japan,^5^ the population peaked in May 2020 and has decreased continuously since then. From July to September 2020, population out-flows continuously exceeded in-flows. Comparing May 2020 and May 2021, the population in Tokyo decreased by about 40,000. Such a large population decline is the first since the end of World War II, showing the magnitude of the influence of the pandemic. In London, the population fell by more than 300,000, indicating the first annual drop since 1988 (Partington, 2021). In both Tokyo and London, the population decline was affected by increases in teleworking during the pandemic. In developing countries, millions of migrants working in cities have been forced to return to their rural homes (FAO, 2021). Such migration has transmitted the COVID-19 virus from cities to rural areas, for example in India (Ghoshal and Jadhav, 2020). Migration has also contributed to the spread of COVID-19 in developed countries. According to a study on Italy (Valsecchi and Durante, 2020), “a 50% increase in potential return migration from outbreak areas relative to the mean is associated with 147 additional total deaths per province.”

#### Employment

The impacts of COVID-19 on employment are tremendous. According to the ILO (2021), working-hour losses in 2020 were equivalent to 255 million full-time jobs, which were approximately four times greater than during the global financial crisis in 2009. The losses are particularly higher in Latin America and the Caribbean, Southern Europe and Southern Asia. Job losses are also serious in developed countries. For example, in the USA, a total of 16 million people lost their jobs as of April 9, 2020, where people out of work were not counted (Rushe and Sainato, 2020), and men, young workers, Hispanics and workers with lower levels of education suffered more than other population groups (Louis-Philippe et al., 2020). The vulnerability of employment to the pandemic seems uneven across regions: for example, employment in Southern Europe and France is more vulnerable than in Northern Europe, while the impacts on employment are moderate in Eastern and Central Europe (Doerr and Gambacorta, 2020). As governments struggle to balance epidemic control measures and economic recovery, the COVID-19 pandemic may have pervasive effects on the way people interact and travel in the future. More specifically, COVID-19 propelled the faster adoption of automation and AI, especially in work arenas with high physical proximity (Lund et al., 2021). These e-activity trends are shaping the future of work by workforce transitions or evolution of the work arenas. As reported by Mckinsey Global Institute (2021), in eight economies, namely the USA, the UK, France, Germany, Spain, China, Japan, and India, more than 100 million workers may need to switch occupations by 2030, a 12% increase from before the epidemic overall, and an increase of up to 25% in advanced countries.

#### Education

As revealed in a report by the United Nations (2020), nearly 1.6 billion students in more than 190 countries were affected by the pandemic, while 94% of the global student population (almost 100% in low and lower-middle income countries) were influenced by closures of schools and other learning facilities. Globally, there are more than 40 million children missing out on basic education at their critical preschool year (UNICEF, 2020b). Such disruptions to the education system is the largest in history. Such an educational crisis poses a global threat to all of the Sustainable Development Goals (SDGs).

As argued by the International Organization for Migration (IOM) (2020), migration matters for recovery from the COVID-19 pandemic because of the high reliance of the economy, food and health on the movement of people. The serious job losses and education disruptions described above suggest the importance of a human-centered recovery from the pandemic.

#### Behavioral nexus

The unprecedented pandemic has led some people to rethink their living environment, which may drive people to relocate to suburbs, smaller cities, second-home destinations and even rural areas (Wang et al., 2020; Zhang et al., 2021). As stated in Zhai and Zhang (2021), teleworking has encouraged some companies to reduce and/or share office space, while more time spent in residential areas may lead to changes in land use patterns in or near residential areas. For example, guaranteeing self-sufficient urban services in or near residential areas for daily life needs may become essential in future urban planning. To guarantee equal access to urban services, spatial planning should be transformed with the support of legal systems; meanwhile, spaces for accommodating urban functions should be well planned and controlled to avoid disorderly development.

### Summary

Interdependencies across life domains suggest the importance of cross-sectoral COVID-19 policymaking. Few existing studies have analyzed all possible life changes from a comprehensive perspective. Lack of research on behavioral co-changes has hindered the reflection of scientific evidence into effective cross-sectoral collaboration in the figtht against the COVID-19 pandemic. Some factors, such as social psychological factors (e.g., subjective norms) (Parady et al., 2020), which are expected to affect multiple behaviors simutaneously, have been ignored. Such ignorance may lead to an underestimation of the impact of the pandemic and poor understanding of the changes in people’s daily lives. Furthermore, existing studies about social contact mainly focus on evaluating the effects of control measures and distancing strategies on virus transmission (Yong et al., 2020; Chiu et al., 2020; Roberts, 2020). To date, there has not been adequate research on interpersonal social contact changes when performing various activities during the pandemic.

## Uncertain trends of people’s lives for the post-pandemic recovery

To answer *“what should be computed?”*, it is necessary to figure out what the post-pandemic recovery will look like, what kinds of recoveries should be expected, and how much can be realized.

**Fig. 2:**
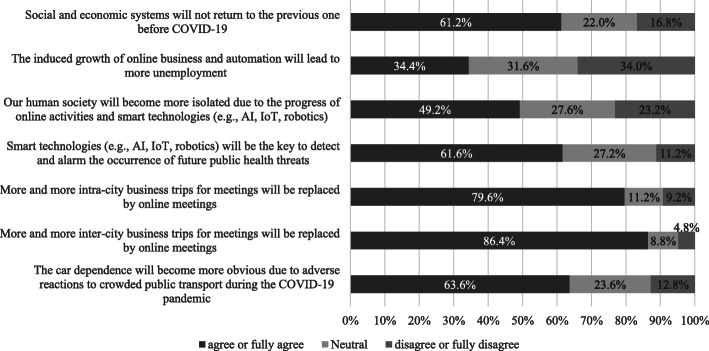
Experts’ opinions about changes in people’s lives caused by the COVID-19 pandemic

According to a worldwide expert survey^6^ conducted by the WCTRS (World Conference on Transport Research Society) COVID-19 Task Force in April and May 2021 (see Fig. 2), 34.4% of experts agreed that the induced growth of online business and automation would lead to more unemployment, while a similar percentage of experts disagreed. Nearly half of the experts surveyed stated that our human society would become more isolated due to the spread of online activities and smart technologies (AI, IoT, robotics, etc.), while experts who disagreed with this statement accounted for 23.2% and the remaining 27.6% showed a neutral opinion. Concerning car dependence, 63.6% of experts predicted that car dependence would become more obvious due to negative reactions to crowded public transport during the COVID-19 pandemic, while only 12.8% disagreed and 23.6% showed a neutral opinion. Online meetings were predicted to replace business meetings involving intra-city trips by 79.6% of experts, while 86.4% predicted that they would replace inter-city trips. Meanwhile, 61.2% of experts thought that social and economic systems would not return to the previous systems before the COVID-19 pandemic, while 22% showed a neutral opinion.

### Concerns about post-pandemic recovery policymaking

How to recover from the current pandemic and how to build our society better than before have dominated the policy agenda in all countries. As observed before the pandemic, digital development has brought about new drivers of economic growth; however, existing social exclusion issues have been (unintentionally) reinforced by the emerging digital divides (van Dijk, 2020). Similar dilemmas may exist with regard to *inclusive recovery*, where digital technologies are expected to play a key role. *Green recovery* after the pandemic may experience similar types of dilemmas observed before the pandemic. However, the urgency and intensity of dilemmas during and after the pandemic may be considerably different. For example, concerns about unemployment caused by promoting green recovery will be more serious. It is also necessary to pay attention to other concerns during the recovery process, such as *moral, resilience, smartness, and quality*, which all involve both co-benefits and dilemmas. *Moral recovery* needs to pay attention to ethical issues (e.g., privacy, human rights, health impacts, altruism) during the recovery, but may slow down the recovery speed (e.g., by bringing more barriers to technology developments) and may also lead to cost-inefficiency (e.g., by taking more time and investing more money in developing technologies). *Resilient recovery* suffers from dilemmas in terms of heavy investments in preparations and lower public acceptance due to cost-inefficiency and invisible effectiveness from a short-term perspective. *Smart recovery* after the pandemic may suffer from similar dilemmas between smart growth and digital divides, as experienced before the pandemic. Dilemmas caused by *quality recovery* may be similar to existing issues related to the quality of development in people’s lives and in cities. Depending on what choices are made to deal with the above concerns, co-benefits may exist in all or part of them. For example, smart technologies can be developed by addressing digital divide issues, while quality recovery challenges may encourage people to make moral decisions. However, it remains unknown whether or not and how the post-pandemic renaissance of the recovery will generate new dilemmas and require new forms of co-benefits.

For example, faced with COVID-19, cities are being called upon to radically change their physical layout and socio-economic structures to cope with the crisis. Meanwhile, in order to build a post-pandemic urban environment that can respond to future health crises, cities are supposed to making new commitments to curb the spread of disease and implement new strategies, actions, rules and planning tools (Kleinman, 2020). The “right to the city” is also the right to reinvent and change the form of the city and the urban environment according to new needs (Harvey, 2012). During COVID-19, cities face the main challenge of guaranteeing a new “right to the city” which not only considers these essential services but also meets a different way of life that is adapted to the new health crisis. It is the right to create a new paradigm of city equity, opportunity and social innovation, which fights inequality, reinforces the sense of community, builds local resilience and sustainability, and aids cities in their recovery from this crisis (Barberis, 2013). This requires that a post-pandemic city should not only return to “normal” but should also contribute to a better, more sustainable and more flexible society. The “new normal” of cities should include formulating new measures to help cities recover and support cities to develop in a more sustainable, low-carbon, inclusive and healthy way. As the Mayor of New York, Bill de Blasio, said, “We need a new deal for these times, a massive transformation that rebuilds lives, promotes equality, and prevents the next economic, health, or climate crisis, and definitively changes the way we live in cities for years to come” (Taylor, 2020).

### Uncertain changes in people’s lives after the pandemic

Will the COVID-19 pandemic end just as a random shock or as a true game changer? How will the COVID-19 pandemic change people’s lives after the pandemic? Will the changes in life observed during the pandemic bounce back? Will people voluntarily change their behaviors to live a greener and healthier life? Will much stronger policies be required to promote a greener and more inclusive recovery? There are many questions to be answered for urban policymaking in the post-pandemic era.

### Will megacities become smaller? A migration perspective

As discussed above, in general there are more COVID-19 infections in megacities than in other cities. If more people are concern about high risks of infections in megacities, the current growth in megacities across the world may slow down, and within a particular country, regional distributions of population may become more balanced. According to the United Nations (2018), there were 1.7 billion people (23%) living in a city with at least 1 million inhabitants in 2018, while the share is predicted to reach 28% in 2030. The population in megacities (10 million) was 529 million (6.9%) in 2018, which is predicted to be 752 million (8.8%) in 2030. Will the current pandemic slow down the growth of megacities? Furthermore, whether will changes in megacities alter the current urban forms or not?

### Will online economy and automation improve or worsen existing social divides?

The current pandemic has resulted in the rapid diffusion of various online businesses. This trend is likely to continue and consequently more delivery transport will occur to replace people’s shopping at stores. Once the economy is re-opened, a rapid growth of automation may also be expected. This suggests that transport and our life as well as our economy will become much smarter. It is worth exploring whether such a development of smart society will help transport systems and services to work in a more sustainable way so that social divides can be minimized.

More and more teleworking and more flexible working arrangements can be expected. This will allow people to work at home and at the same time to take care of children, without seriously affecting their work and generating any negative transport impacts. However, not everyone can telework, because of the type of work they do, access to the Internet and availability of telework tools, restrictions of institutions, house conditions, etc. Thus, better support to reduce potential social divides is needed, such as offering a better work environment in houses through upgraded equipment, reliable and safe internet, and synchronized workload management.

### Will digital development (including teleworking) change urban forms? A perspective on residential and travel behavior

Teleworking has proved that it can assist people to save time spent on commuting. Long stays at home during the pandemic has raised questions about how to live a better life in residential areas. Active transport has become more popular during the pandemic, providing a unique opportunity to think about how to re-allocate road spaces to different types of traffic. All these trends may encourage people to re-think issues around housing and job proximity, which involves a variety of co-benefits. If time spent in residential areas increases considerably, people will expect more functions to support their lives, which will attract more firms and other daily facilities and services. Accordingly, changes in urban forms can be expected, which are probably further influential to changes in CO2 emissions. However, people’s resistance to these changes should not be ignored.

### Will CO2 emissions be reduced faster or remain unchanged?

As stated previously, global CO2 emissions decreased in the first half of 2020 but had bounced back close to 2019 levels by the end of 2020. Will CO2 emissions be reduced faster or remain unchanged due to changes in people’s lives? Will people become more pro-environment? How to make use of big data to support people’s environmentally friendly behavior changes?

After the pandemic, a ‘new normal’ is expected due to dramatic changes in people’s lifestyles. The current experience with bluer skies may trigger people’s pro-environmental attitudes. If this is the case, energy consumption from both in-home and out-of-home activities will decrease. It is, however, not clear whether such dramatic changes in lifestyles will result in more or less household energy consumption in general. If more people shift to telework-centered workstyles, obviously, energy consumption from commuting will be dramatically reduced; however, it is not obvious whether the reduction in commuting will be cancelled out by more in-home energy consumption or not.

### Will people pay more attention to planetary health?

Because of longer periods of staying at home, people have been advised via various channels about how to maintain their health, especially how to do physical exercise at home and eat in a healthy way. More and more people are following these instructions about healthy lifestyles. Related to travel behavior, active travel has been attracting attention, while increases in car dependency is also expected to occur at the same time. It is therefore unclear whether people’s health conditions can be improved via active travel or not. This needs more careful research. Long stays at home during the COVID-19 pandemic have also induced a lot of stress in some people. Such stress may be released suddenly after the pandemic is over, for example through travel and tourism for relaxation.

According to Hinchliffe et al. (2021), the COVID-19 pandemic and other public health threats are linked with habitat destruction, illegal trade in wild animals, climate instabilities, and changing intensities in the relationships between humans and other animals, while the relevance of race, poverty, identity, and violent racism in unequal health outcomes has complicated these links. Human health has been improved by sacrificing the degradation of nature’s ecological systems.^7^ In recent years, planetary health has been attracting more and more international attention. It refers to “the health of human civilization and the state of the natural systems on which it depends”, meaning “the achievement of the highest attainable standard of health, wellbeing, and equity worldwide” (Whitmee et al., 2015). The concept of planetary health postulates that human civilization, human health, natural systems and resources are inextricably linked.^8^ It characterizes “the human health impacts of human-caused disruptions of Earth’s natural systems.”^9^ As a new concept related to health, planetary health can be interpreted as total health of human, society, and nature. All the above-discussed uncertainty issues are within the scope of planetary health. CO2 emissions are a key concern of planetary health. It is crucial to figure out whether changes in urban forms will lead to healthy or unhealthy interactions between human and nature. If the popular digital development will cause more social divides, then digital development should be better regulated for healthier social development. However, it remains unclear whether people will pay more attention to planetary health or not from various angles in the future.

## Conclusion: what should be computed?

The COVID-19 pandemic is still ongoing, and it is unknown when it will come under control. Based on a review of the literature, it is found that the impacts of COVID-19 on people’s lives are tremendous and diverse, the revealed associations between the spread of COVID-19 and people’s mobility are inconsistent, and the post-pandemic changes are still uncertain. *Computable Urban Science* should help clarify these unknowns, where big data analyses are expected to play a key role. In this regard, it is necessary to pay attention to the so-called ecological correlation (fallacy): i.e., a correlation between two variables that are group means is not necessarily equal to a correlation between two variables at the individual level. In other words, findings based on aggregated big data are not necessarily applicable to the individual level. The post-pandemic recovery needs to be supported by people’s lifestyle transformations, which refers to changes in behaviors at the individual level. This suggests the importance of making use of big data at the individual level. Big data is rich in information about behavioral outcomes; however, it is poor in information about decision-making processes and influencing factors. This further suggests that it is worth integrating (large-scale) big data with rich outcome information on the one hand, and (small-scale) questionnaire data with rich information about process and factors on the other.

### Computing data-driven solutions to overcome delays in the SDGs caused by the COVID-19 pandemic

The COVID-19 pandemic has brought out new challenges to the realization of the UN’s Sustainable Development Goals (SDGs), whose target year is 2030. Within just a short period, the pandemic has caused the worst economic resession (GDP growth: − 4.2% in 2020) since the Great Depression, worsening progress in achieving the SDGs, especially in terms of poverty, hunger, unemployment, health care and education.^10^*Computational Urban Science* can and should contribute to developing data-driven solutions on how to overcome delays in the SDGs caused by the pandemic. The SDGs include 17 goals and 169 targets, which are connected with each other (Shulla et al., 2021). Solutions making proper use of such interconnectedness are expected to accelerate progress in meeting the SDG targets and generate co-benefits or synergetic effects; however, poor recognition of this interconnectedness will bring more barriers to the SDGs. Big data supporting the SDGs is unevenly distributed across countries and cities. It is therefore important to carry out research on the transferability of data-driven solutions from countries and cities with sufficient big data to those without sufficient data.

### Computing to address digital divides and dilemmas of e-society

The current COVID-19 pandemic has triggered the further development of the online platform economy or simply platform economy, which has helped people to survive from the various impacts caused by the pandemic (Bekarmino et al., 2021; Gerwe, 2021). More and more people have become familiar with e-activities, which have penetrated across our society, widely and deeply. Such a society supported by e-activities is also called e-society. E-government or e-governance, e-business or e-commerce, e-learning or e-education, e-health, e-dating, e-sports, e-tourism, and e-leisure are all within the e-society domain. We may name our life supported by e-activities as e-life.

However, not everybody can benefit equally from such online services. Digital divides and delimmas have existed since the birth of the Internet. Root causes of the digital divide include the lack of digital infrastructure and services; lack of affordable network services, devices and applications; lack of digital capacity (knowledge and skills, etc.) to understand contents and create/add value; demographic/cultural causes (e.g., aging population on the rise, socially excluded population groups (e.g., women)); locational disadvantages (e.g., rural areas); lack of adapted devices and websites for differently abled users (disability); and lack of coordinated efforts to foster social and economic equality, etc. Age, gender, ethnicity, labor, education and nation or region are the most important factors in explaining digital inequality in all access phases (motivation attitude, physical access, digital skills, and usage).

Despite the existence of the digital divides and delimmas, various on-demand information platforms have offered more and more new job opportunities. It is therefore necessary to examine the impacts of COVID-19 on digital labor platforms, such as freelance online web-based platforms and location-based platforms (transportation and delivery platforms), which have grown exponentially over the past decade. One of the inclusive recovery strategies may be the promotion of the sharing economy. Emerging crowd-shipping information platforms not only provide people convenience in sending and receiving packages, they also offer job opportunities. In principle, everyone deserves the equal opportunity to participate in crowd-shipping platforms as a part-time or full-time job. The participation may be especially beneficial to unemployed people and residents in rural areas who were greatly affected by the COVID-19 pandemic. In addition, the income of participating package delivery may not be as high as other jobs. Obvious heterogeneity in the preference of different people can be expected, especially when taking into account their own personality, attitudes and social influences. Before including participation in package delivery as one of the inclusive recovery strategies, it is necessary to gain more insights into people’s participation behavior. Existing work about participating in the sharing economy in transport mainly addresses decisions concerning ride-sharing participation, while participation in crowd-shipping, especially from the perspective of the labor market, remains underresearched.

As evidenced by van Dijk (2020), digital inequality has reinforced existing social inequality over the years. During the current pandemic, for example, teleworking has generated disparities between regular and non-regular employment in Japan, and the lockdown strategies in the UK are actually increasing digital inequality. Children in households without an Internet connection cannot participate in online classes, while people who cannot access the Internet are not able to learn skills or offer skills via online platforms during the pandemic. Elderly and low-income people, as well as those without sufficient information literacy, are vulnerable to digital divides. The digital divides have been a key factor causing educational, economic and social disparities.

### Computing to capture behavioral co-changes during the post-pandemic recovery process

To recover from the COVID-19 pandemic, it is necessary to design policies which direct people’s behaviors towards resilient and sustainable future. However, behavioral changes involve complicated decision-making mechanisms, which have not been well understood. If a change in a certain behavior is constrained by other behaviors, the change may take place only when changes in other behaviors occur. For example, in the context of domestic migration, Zhang et al. (2017) revealed that the intention of migration is strongly affected by intentions to change jobs, dwellings, and child rearing. In this sense, it is important to pay more attention to behavioral co-changes in multiple life domains during the post-pandemic recovery process. Taking the green recovery as an example, promoting pro-environmental lifestyles is important. In this context, in order to change people’s car use behaviors, it is necessary to first unfreeze travel behavior habits; however, this is a difficult task. A pro-environmental trigger changing people’s use of air-conditioners at home may be helpful to unfreeze travel behavior habits. Such a trigger may also have a co-benefit of health. Decisions in different life domains usually have different time windows. Thus, the analysis of behavioral co-changes in multiple life domains needs to properly reflect such differences of decision-making time windows. In the beginning of this section, the importance of integrating big data and questionnaire data is highlighted. In the same way, the aforementioned data integration should properly reflect behavioral interdependencies across multiple life domains. Exploring behavioral co-changes is beneficial for cross-sectoral policymaking and therefore the post-pandemic recovery.

The behavioral co-changes described above are due to various interactions across life choices (e.g., residence, employment, education, household life, travel behavior, expenditure, time use, health, energy) in various time scales (short-term, medium-term, long-term, and the life course). Convincing approaches which capture both spatial and temporal dynamics of individual-level behavioral changes in response to policy interventions and which can measure aggregated effects of individual changes at the city/regional level are still limited. It seems possible to first integrate the processes of multiple behavioral changes with various interrelated behavior outcomes at the individual/household level and then aggregate all individual−/household-level processes and outcomes at city−/region-level scales.

### Computing to address methodological challenges

The inconsistent evidence about the spread of COVID-19 and associated factors discussed earlier is partially due to methodological issues. For example, an important methodological issue which has largely been ignored was whether the impact of density may depend on the spatial scale of the geographic analytical units (e.g., counties, city areas, or census blocks) used in analyzing the correlation between density and infection of COVID-19 (Huang et al., 2020). This is the well-known Modifiable Areal Unit Problem (MAUP) (Dark and Bram, 2007). It means that research findings may vary due to different spatial scales or zonal schemes of the geographic areas being used to derive the area-based variables (e.g., city/county density and infection rates). Spatial heterogeneity of infection has been confirmed. For example, Ma et al. (2021) adopted a random forest approach and analyzed partial dependence plots by focusing on COVID-19 infections and potential factors measured at the township-level. They revealed various nonlinear associations between the spread of COVID-19, various built environment indicators and population mobility, where built environment indicators were used as a proxy for interpersonal communications or contacts. The investigation of more precise transmission processes in confined spaces should be considered, and it is worth exploring how safe a densely packed space is for a population.

### Computing for process management of post-pandemic recovery policymaking

Recovery policies should be implemented based on proper process management. However, there is a serious lack of research on this type ofmanagement. Transformative thinking is required for both controlling the current pandemic and also promoting the post-pandemic recovery. Zhang (2021b) proposed a six-step DIRECT approach, including *Detect*, *Inform–Intervene*, *React*, *Enlighten–Enforce–Evaluate*, *Collaborate*, and *Transfer*. For example, for the inclusive recovery from the perspective of employment, policymakers should *detect* (**D**) unemployment issues or those people at risk of unemployment in a timely manner, and accordingly, *inform* (**I**) them about governmental support and employment opportunities. Intervening (**I**) in the job hunting process may be needed (e.g., providing advice and job training), depending on actual situations. These target people may or may not react (**R**) to the informed interventions. In such a case, “enlighten/enforce” (**E**) will be needed. “Enforce” does not mean a forced employment; rather, it means that the informed interventions need to be enforced to better help those people. In this regard, all of the above four steps need to be properly evaluated (**E**), especially for getting broad and effective collaboration (**C**) from more governmental departments, firms, communities, and other stakeholders. Such efforts should be better *transferred* (**T**) to other employment issues. To realize a greeen recovery, it is crucial to *detect* (**D**) high-carbon energy consumption patterns in buildings and transport in a timely way, thereby avoiding an unnecessary waste of energy. People inside buildings and trip makers may not recognize their high-carbon energy consumption behaviors, suggesting the necessity of better informing and/or intervening in them (**I**). Considering the existence of rebound effects of energy consumption, energy-saving technologies may not necessarily guarantee energy reduction, as expected. This means that “enlighten/enforce” (**E**) will be needed. “Enforce” does not mean a forced reaction, but rather that the informed interventions need to be enforced in order to enhance the intervening effects. Similarly, all of the above D-I-R-E steps need to be properly evaluated (**E**) with respect to the involvement of governmental departments, firms, communities, and other stakeholders in deploying broad and effective collaboration (**C**) in energy-saving. Such efforts also need to be better *transferred* (**T**) to other sustainable issues.

## Data Availability

Not applicable.

## Footnotes

1 As of August 10, 2021, there were 516,785 cumulative infections and 13,068 deaths, according to Worldometers (https://www.worldometers.info/coronavirus/country/greece/)

2 https://eleminist.com/article/1254 (in Japanese; Accessed on July 31, 2021)

3 https://www.iea.org/news/global-energy-demand-to-plunge-this-year-as-a-result-of-the-biggest-shock-since-the-second-world-war [Accessed on May 2, 2020]

4 https://www.businessinsider.com/countries-on-lockdown-coronavirus-italy-2020-3 [Accessed on May 2, 2020]

5 https://www.toukei.metro.tokyo.lg.jp/jsuikei/js-index2.htm

6 The website is purposely made unaccessible. This will be accessible if the paper is accepted.

7 https://www.thelancet.com/commissions/planetary-health

8 https://unfccc.int/climate-action/momentum-for-change/planetary-health

9 https://www.planetaryhealthalliance.org/planetary-health

10 https://www.un.org/development/desa/dpad/publication/un-desa-policy-brief-81-impact-of-covid-19-on-sdg-progress-a-statistical-perspective/
